# Future large hydropower dams impact global freshwater megafauna

**DOI:** 10.1038/s41598-019-54980-8

**Published:** 2019-12-06

**Authors:** Christiane Zarfl, Jürgen Berlekamp, Fengzhi He, Sonja C. Jähnig, William Darwall, Klement Tockner

**Affiliations:** 1 Center for Applied Geoscience, Eberhard Karls University of Tübingen, Hölderlinstr. 12, 72074 Tübingen, Germany; 2 Institute of Environmental Systems Research, University of Osnabrück, Barbarastraße 12, 49076 Osnabrück, Germany; 3 Department of Ecosystem Research, Leibniz-Institute of Freshwater Ecology and Inland Fisheries, Müggelseedamm 310, 12587 Berlin, Germany; 4 Department of Biology, Chemistry and Pharmacy, Freie Universität Berlin, Altensteinstraße 6, 14195 Berlin, Germany; 5 School of Geography, Queen Mary University of London, London, E1 4NS UK; 6 Freshwater Biodiversity Unit, IUCN Global Species Programme, The David Attenborough Building, Pembroke Street, Cambridge, CB2 3QZ United Kingdom; 7 Austrian Science Fund, Sensengasse 1, 1090 Vienna, Austria

**Keywords:** Freshwater ecology, Biodiversity, Environmental impact

## Abstract

Dam construction comes with severe social, economic and ecological impacts. From an ecological point of view, habitat types are altered and biodiversity is lost. Thus, to identify areas that deserve major attention for conservation, existing and planned locations for (hydropower) dams were overlapped, at global extent, with the contemporary distribution of freshwater megafauna species with consideration of their respective threat status. Hydropower development will disproportionately impact areas of high freshwater megafauna richness in South America, South and East Asia, and the Balkan region. Sub-catchments with a high share of threatened species are considered to be most vulnerable; these are located in Central America, Southeast Asia and in the regions of the Black and Caspian Sea. Based on this approach, planned dam locations are classified according to their potential impact on freshwater megafauna species at different spatial scales, attention to potential conflicts between climate mitigation and biodiversity conservation are highlighted, and priorities for freshwater management are recommended.

## Introduction

Many economies in the Global South are booming, and electricity demand is rising rapidly. At the same time, 940 million people do not have access to electricity (2016), especially in low-income regions^1^. Therefore, access to “affordable, reliable, sustainable and contemporary energy” is one of the 17 UN Sustainable Development Goals (SDG 7), which came into force in 2016. At the same time, immediate measures are required to “combat climate change and its effects” (SDG 13) and to sustainably manage terrestrial ecosystems, where “terrestrial” includes inland waters (SDG 15). In concrete terms, the Paris Agreement, to which all states (except Syria, and recently also the USA) have been parties since November 2017, aims to reduce greenhouse gas emissions, which is envisaged by 157 countries primarily in the energy sector^2^. Climate mitigation strategies promote the expansion of so-called “climate-neutral” electricity sources.

Dam construction is an established technology to generate hydropower, which is a renewable albeit not climate-neutral electricity source: Currently, 22% of the world’s electricity is provided by renewable resources, 73% of which by hydropower^1^. Following the United Nations Climate Change Conference, 21^st^ Conference of the Parties (COP21) in Paris in 2015, many governments decided to include the expansion of hydropower construction within their “Intended Nationally Determined Contributions” to address climate change. Indeed, more than 3,700 medium and large hydropower dams are under construction or planned worldwide (henceforth called “future” hydropower dams), which will double the currently installed capacity^3^. Even if some of these plans may have long been neglected, the actual “window-of-opportunity” might bring them back to life.

The global boom in hydropower dam construction will mainly take place in South America (Amazon and La Plata River basins) and in South and East Asia (Ganges-Brahmaputra and Yangtze basins) as well as in Africa, which will be experiencing a specifically strong relative increase due to its high remaining technical hydropower potential. In Europe, the focus will be on the Balkan region with more than 600 future hydropower plants, either planned or under construction, each with a capacity >1 MW^3^. If smaller hydropower plants were included, there would be more than 3,000 plants constructed in this region^4^.

Since hydropower reservoirs can be used to store energy and generate it when required, the increase in hydropower capacity can, at the same time, support the expansion of further renewable energy technologies when installed in combination with hydropower. Hence, hydropower increases the security of energy supply in an integrated electricity market in which solar and wind technologies are being expanded^5^. In addition, hydropower plants are often not only used to generate electricity, but impounded reservoirs serve the human population as recreational areas, flood protection, for aquaculture, drinking water or for irrigation purposes^5^. Hence, the expansion of hydropower is considered a potential solution to multiple challenges.

At the same time, the construction and operation of power plants have their downsides, in part with serious and long-term effects^6^. Negative consequences include social impacts on local human communities and cross-border conflicts regarding water use and availability^7^, changes in hydrology and sediment transport^8,9^, greenhouse gas emissions due to degradation of accumulated organic materials under anoxic conditions^10^, deterioration in water quality, e.g. due to toxic cyanobacterial blooms^11^, spread of water-associated diseases and invasive species^12,13^, as well as changes in habitat conditions, fragmentation of fish migration pathways, loss of biodiversity and erosion of ecosystem services^8,14–16^. Hence, the expected boom in dam construction undermines a number of the SDGs and the Aichi biodiversity goals (Target 12), formulated in 2010 as part of the Nagoya Protocol, to implement the goals of the UN Convention on Biodiversity. Goals, for example, include the significant reduction of natural habitat loss (Target 5), maintenance of a high degree of connectivity, and prevention of species extinctions along with their improved status (Target 12)^17^.

Freshwaters are among the most diverse ecosystems globally. While only 2.3% of the earth’s terrestrial surface is covered by rivers, lakes and reservoirs, freshwaters are habitats to about 9.5% of known animal species^15^. Freshwater megafauna species (≥30 kg)^18^ serve as potential surrogates for evaluating the status of all freshwater biodiversity; consequently, the loss of these charismatic species could indicate a loss of co-occurring smaller, less visible species^19^. Indeed, the impact of dams is reported as one of the biggest pressures on freshwater megafauna^20^. For example, dams block migration routes for megafishes such as sturgeons and giant catfish, reduce access to fish spawning areas, and cause habitat loss and degradation in both downstream and upstream reaches^21^. Together with overexploitation and bycatch, dams have played a significant role in range contraction and population decline of several freshwater megafauna species including the Indus river dolphin, Chinese paddlefish and several sturgeon species (e.g. the beluga, Chinese sturgeon, Yangtze sturgeon, Russian sturgeon)^22,23^.

This study focuses on three main research questions: (1) To what extent may future hydropower dams affect the distribution ranges of freshwater megafauna on the global scale? (2) In which river basins will the threat to megafauna species by future dams be greatest? (3) Will those sub-catchments supporting the highest species richness and share of threatened megafauna species be disproportionately affected by future hydropower construction? The results of these analyses will highlight any potential conflicts between climate mitigation and biodiversity conservation, and help set priorities for future management of freshwater ecosystems.

## Results

### Spatial overlap between future hydropower dams and sub-catchments rich in species of freshwater megafauna

On the global scale, river basins with the highest richness in species of freshwater megafauna show high levels of congruence with locations for proposed hydropower dams (>1 MW in capacity; Fig. S1). The Amazon, Mekong and Congo basins are especially rich in megafauna species, as are the Orinoco and the Ganges-Brahmaputra basins^18,20^. At the same time the Ganges-Brahmaputra (396 future dams, total capacity 41 GW), Amazon (368, 170 GW) and Mekong (120, 40 GW) basins are among those with the highest number of hydropower dams under construction or planned. Similarly, dam construction plans in the Congo basin are expected to add 44 GW of hydropower (35 future dams). The highest numbers of proposed dams are for the La Plata basin in South America (411 future dams, 20 GW), and the Danube basin in Europe (342 future dams, 10 GW), with similarly high numbers proposed as for the Amazon. The number of future hydropower dams in the entire Balkan region, including the Danube basin and the basins of the Adriatic Sea/Greece/Black Sea Coast, is particularly high (608 dams, 17% of the global total number).

**Figure 1:**
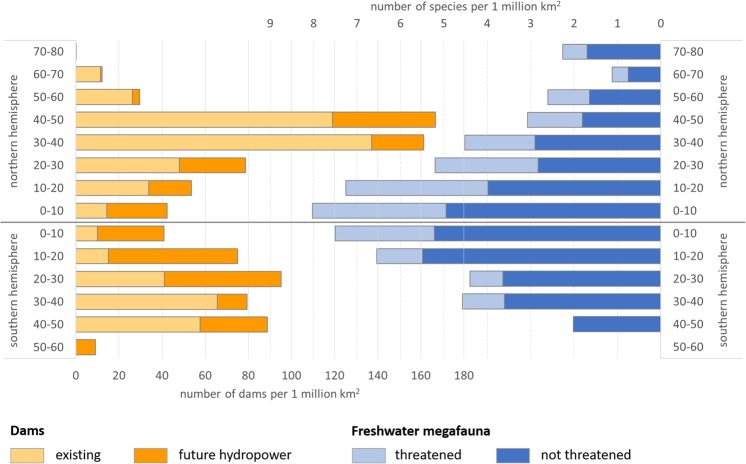
Number of existing dams and future hydropower dams (left) and freshwater megafauna species, threatened and not threatened, (right) along latitudes.

There will be a major geographic shift in the location of hydropower dams, from existing dams (>15 m in dam height) in temperate latitudes (30–60°) to future hydropower dams in tropical to subtropical latitudes (<30°), especially on the southern hemisphere (10°S–30°S, Fig. 1). This will result in a more even distribution of dams across latitudes (both hemispheres). The majority of future dams (64%) will be built in latitudes rich in species of freshwater megafauna, i.e. in latitudes with 5 to 8 megafauna species per million km^2^. The proportion of threatened species is highest in the northern hemisphere with more than 25% of species threatened across all northern latitudes where freshwater megafauna species occur (Fig. 1).

**Figure 2:**
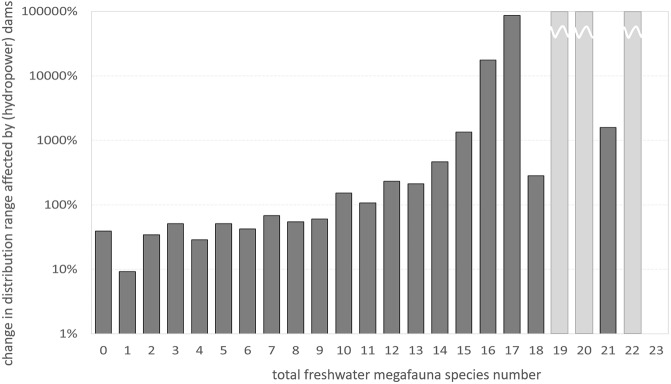
Relative change in freshwater megafauna distribution range affected by hydropower dams in the future compared to distribution range affected by existing dams on a sub-catchment scale (compare Fig. 1 for latitudinal scale). Please note: The y-axis is given in log scale; there are no existing dams but future hydropower dams in sub-catchments with a number of 19, 20 or 22 megafauna species (light grey columns), thus, mathematically the relative change cannot be calculated but converges to infinity. Absolute values for distribution range fractions affected by existing dams and future hydropower dams can be found in Fig. S3.

The majority of future dams is planned in species-rich geographic latitudes (i.e. tropics and sub-tropics) as well as in species-rich river sub-catchments (HydroBASINS level 8, Fig. S3). While existing dams are mainly located in sub-catchments inhabited by up to 15 megafauna species, future hydropower dams will dominate in sub-catchments with 10–23 species. The change in distribution ranges that are directly affected by future hydropower dam construction is thus the most pronounced for the richest sub-catchments (Fig. 2). Fractions of the distribution range that might be directly affected might seem to be small but this number serves as a relative indicator to compare different catchments and sub-catchments.

### Overlap of dams with freshwater megafauna distribution ranges rich in species and with a high share in threatened species

**Figure 3:**
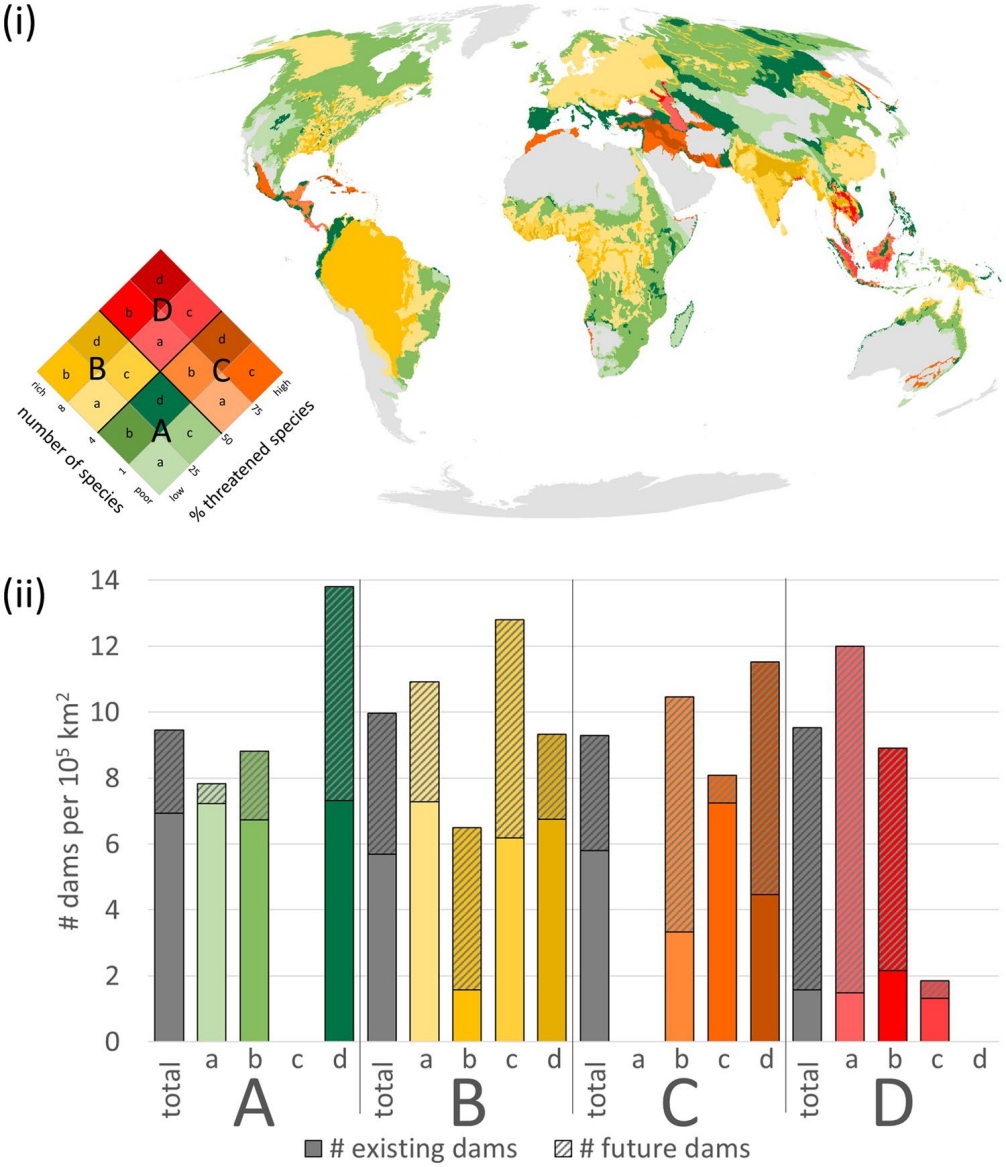
(**i**) Choropleth map of sub-catchments (HydroBASINS level 8) according to species richness and threat status on a global scale (species presence reference scenario). (**ii**) “Density” of dams per 10^5^ km^2^ in different sub-catchments according to richness-threat categories. (A) green: low richness (≤4 species), low share in threatened species (≤50%); (B) yellow: high richness (>4 species), low share in threatened species; (**C**) orange: low richness, high share in threatened species (>50%); (D) red: high richness, high share in threatened species. Existing dams: bold colour; Future hydropower dams: striped colour.

In addition to freshwater megafauna species richness the share in threatened species provides an indication for the potential vulnerability of the respective sub-catchment. Sub-catchments with a high megafauna species richness and a high share of threatened species (category D, Fig. 3) can be found in river basins in Central America, in the Black Sea, the Caspian Sea and in Southeast Asia. Among existing dams, 32 fall into category D, while 162 hydropower dams (21 GW) under construction or planned overlap with the most sensitive sub-catchments. The most vulnerable major basins of category D are located in the “Black Sea, South Coast” (89 future hydropower dams, 4.1 GW), the Southern Central America basin (35 dams, 0.25 GW), and the Mekong (30 dams, 15 GW), but also the major basin “Viet Nam Coast” (4 dams, 66 MW), the Sumatra basin (2 dams, 0.1 GW), the North Borneo Coast (1 dam, 1.3 GW) and the Danube (1 dam, 42 MW) (Fig. S5). For comparison, sub-catchments with low species numbers and low share in threatened species (category A) contain 3,729 dams and will receive 1,358 additional hydropower dams (262 GW) while sub-catchments with a high species richness and a low share in threatened species (category B) already contain 2,527 existing dams and expect 1,894 hydropower dams more (464 GW). Nevertheless, in terms of dam density, i.e. relating the dam number to the total area of the respective category, the biggest change in dam construction (by about 500%) can be expected for sub-catchments that are characterized by high species richness and a high share in threatened species (category D; Fig. 3ii).

### Ranking of future hydropower dams on the major basin scale according to their location

**Figure 4:**
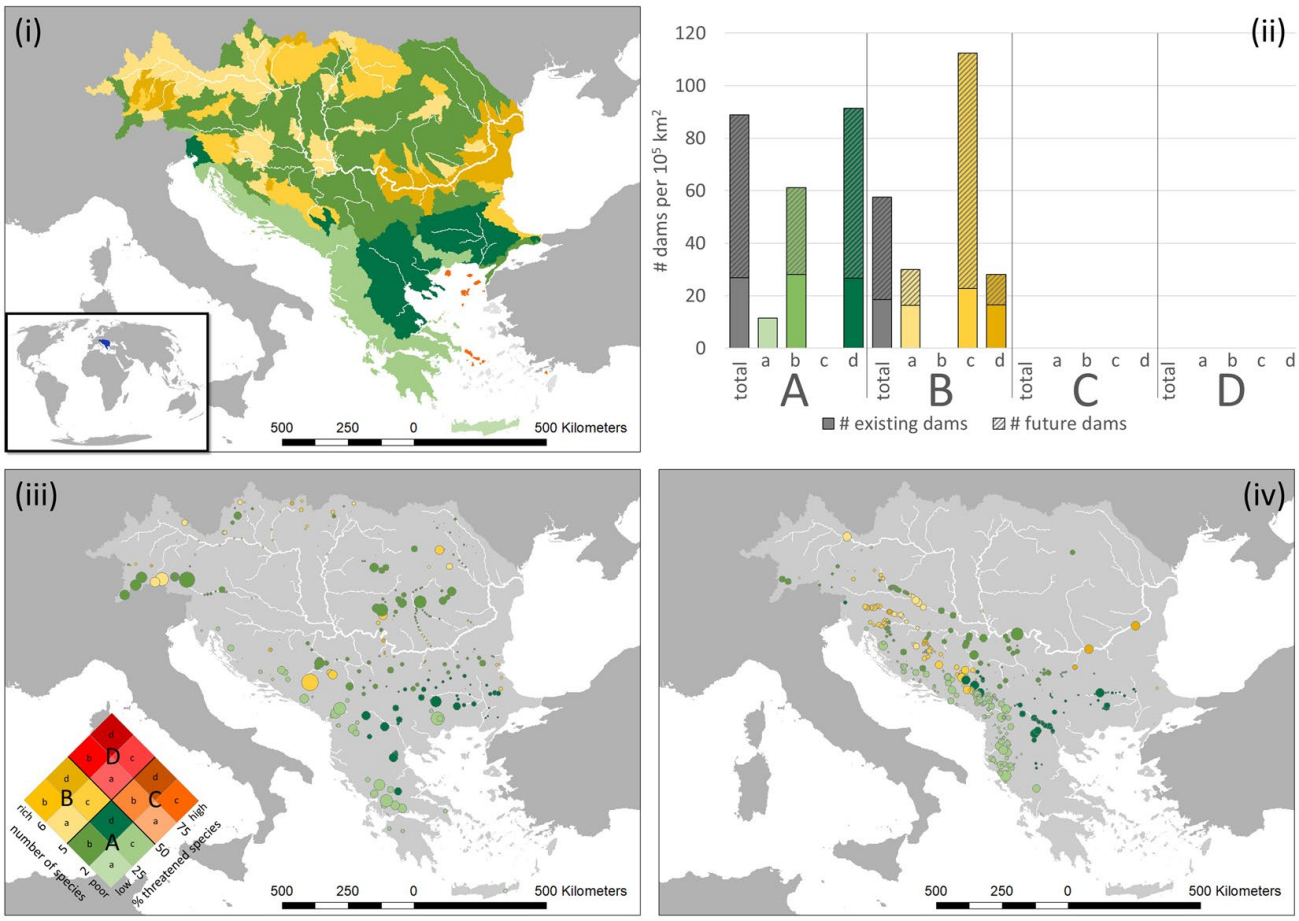
Overlap of dams with freshwater megafauna species richness and share in threatened species for the Balkan area. Top: (**i**) Choropleth map of sub-catchments (HydroBASINS level 8) according to species richness and threat status (species presence reference scenario). (**ii**) “Density” of dams per 10^5^ km^2^ in different sub-catchments according to richness-threat categories. Existing dams: bold colour; Future hydropower dams: striped colour. Bottom: Location of existing dams (**iii**) and future hydropower dams (**iv**) and their individual categorization according to species richness and proportion of threatened species. Size of the dots indicates size of the dam (existing: dam height in m; future: capacity in MW – not scaled). Colour code: Green (A): low richness (≤5 species), low share in threatened species (≤50%); Yellow (B): high richness (>5 species), low share in threatened species; Orange (C): low richness, high share in threatened species (>50%); Red (D): high richness, high share in threatened species. For more details, please see legend in subfigure (**iii**).

Future dams are mainly located in sub-catchments across the full range of categories of species richness and a share of less than 50% species threatened (Figs. 4 and S6). The only exception is the Mekong basin with an increase in hydropower dams also in sub-catchments with a share in threatened species between 50% and 75%. The Yangtze basin is characterized by a large number of existing dams while the other basins are dominated by a high share in future hydropower dams.

Comparing the major basins selected, the “density” of existing and future hydropower dams per richness-threat category shows a large variation from a maximum of 3.7 dams per 100,000 km^2^ in the Congo basin (category: 5–9 freshwater megafauna species, 25–50% threatened) and 112 dams per 100,000 km^2^ in the Balkan basin (category: 5–6 freshwater megafauna species, 25–50% threatened). In terms of dam numbers in relation to available basin area, the Balkan region is thus the region that experiences the largest boom in hydropower dam construction in sub-catchments that are relatively rich in freshwater megafauna species and characterized by a large share in threatened species (25–50%). Thus, the global analysis provides information on potential “priority” sub-catchments in terms of overlaps between dams and freshwater megafauna distribution ranges that are rich in species numbers and have a high share in threatened species.

## Discussion

### Global dam construction and biodiversity

This is the first study exploring the potential impact of future dams, on a global scale, on freshwater megafauna – as a surrogate indicator for total freshwater biodiversity. Recent studies demonstrated that freshwater biodiversity is much more threatened than that in terrestrial and marine environments, with hydropower dam construction as a key underlying driver of biodiversity threat^15^. Hence, the global analyses on the spatial congruence between dam locations and freshwater megafauna species richness, and the respective share of threatened species, help to identify regions that might be most sensitive to dam constructions.

Our results demonstrate that the construction of large future hydropower dams will particularly affect species-rich catchments located in the subtropics and tropics. This can be attributed to the large hydropower potential still available in emerging economies to support industry and development, while much of the respective and easily accessible hydropower potential is already exploited in industrialized countries^24^. Nevertheless, many small hydropower dams are planned in less potential areas but data are not consistently available on a global scale to complement this analysis.

Freshwater megafauna are potentially useful surrogates of overall freshwater biodiversity^19^ as also highlighted for fish species in the Amazon, the Congo and the Mekong basins by Winemiller *et al*.^16^. This means that a high freshwater megafauna species richness implies high overall freshwater biodiversity. Areas with low freshwater megafauna species richness, in contrast, could still have a high overall freshwater biodiversity since megafauna species, due to their habitat requirements and slow life history, are specifically susceptible to extinction^25^. The Amazon, the Mekong and the Congo basins will also experience a major boom in hydropower dam construction, which already has raised major scientific and public attention^26,27^. In Europe, the Balkan region is a hotspot of freshwater biodiversity as well as of future hydropower dam construction that even outnumbers other major basins in terms of dam density based on available data. Thus, there is an urgent need to balance biodiversity conservation with dam development in order to fulfil international agreements, including the European Water Framework Directive, the SDGs (e.g. SDG 6 on restoration of freshwater ecosystems and SDG 15 on species conservation) and the Aichi biodiversity goals.

Globally, hydropower dam construction will most likely increase the pressure on freshwater megafauna – even more than demonstrated, because our rather conservative analyses do not consider impacts of alterations in the flow, sediment and temperature regime downstream of dams^28,29^. For example, the reproduction of the Chinese sturgeon is most likely delayed and reduced due to an altered thermal regime caused by dams^30,31^. Entrapment of sediments in the reservoir may propagate downstream to the delta region and change the morphology within the river channel and the delta^32^. Also, river fragmentation impedes species migration up- and downstream as well as transport of nutrients downstream^33–35^. Changes in the natural flow and flood dynamics affect lateral connectivity between the channel and the riparian zone. These alterations are very site-specific and depend on the topography, climate and other catchment characteristics. Thus, on the global scale, potential longitudinal and lateral impacts of dams on freshwater megafauna were beyond this study, especially considering the limited knowledge on migratory routes and spawning and nursing grounds of megafishes.

Here, we focus on local impacts of dam construction. Hence, the overlap indicates a **potential** impact on megafauna species richness. Several studies confirm that dam building threatens biodiversity^30,36^. Nevertheless, a high share of threatened freshwater megafauna species in a sub-catchment with existing dams is not necessarily a consequence of the dams. This is also true the other way around: If a future hydropower dam is planned in a sub-catchment categorized by high megafauna species richness this does neither necessarily imply that the number of megafauna species will decline nor that it will be unaffected due to this dam construction. The actual correlation depends on several factors: the species itself and its specific traits which might be more or less sensitive to the ecosystem changes, the species capacities to move to another suitable habitat within that sub-catchment and the longitudinal accessibility of it, and dam operation. In addition, “secondary” impacts of dam construction play a role, e.g. further infrastructure development, human settlement and changes in land use that might increase the pressure on that species. A historic decrease in freshwater megafauna due to existing dams has not been analysed here, but data on historic distribution ranges in regions of Europe and the USA is available from IUCN and NatureServe and has been analysed by He *et al*.^23^. The current distribution ranges have been used in this study as reference state for scenarios on future dam construction.

Freshwater megafauna species are specifically threatened by pressures like fishing and hunting, habitat degradation and pollution^20,37^. Nevertheless, out of the 207 freshwater megafauna species investigated, distribution ranges of 191 species overlap with dams (35 with existing dams only, 17 with future hydropower dams only and 139 with both). These overlaps are very specific for the single major basins and differ in dam density relative to the respective habitat range of the species and in the share of threatened species that are (potentially) affected by dam construction (Fig. S7 for details on selected major basins). While interactions of a dam with fish species seems obvious, and is discussed in many studies (e.g. Liermann *et al*., Winemiller *et al*.)^16,21^, impacts on amphibians, reptiles and mammals that spend an essential part of their lifetime in freshwater are often neglected. Also, species that require wetlands as habitats or hunting ground (like crocodiles) as well as species that, in turn, depend on specific freshwater species might directly or indirectly be affected by dam construction. Thus, in considering freshwater megafauna as surrogates this study approximates potential impacts of dams on freshwater biodiversity in general as a potential “net change” without to distinguish between those species that might decline or need to migrate due to dam construction and those that might benefit from reservoir formation.

### Prioritization of future dam locations on a basin scale

Scale and resolution play a crucial role in presenting and interpreting results in areas that might be potentially impacted by future hydropower dams. The analysis on a major basin scale provides, in comparison to the global scale, more spatially-explicit details for decision makers about potential impacts of specific dam locations. Successful river basin management means that the entire river network must be taken into account. The present study allows identifying future dam locations within the respective basin that might have the biggest impact in terms of overlap with species numbers and a high share in threatened species. Categorization is based on relative values within the sub-catchments and basins and not on absolute species numbers, which facilitate comparison among basins (similar to discharge indicators). The (sub-)catchment scale is a “natural” scale that is determined by the structure of the river system. Nevertheless, several countries also share river systems and need to co-operate if the shared resource water is to be impacted by dam building activities up- or downstream of the own country. This is worthwhile, especially as a cross-border approach to identifying the best possible locations for future dams promises higher returns for investors^38^. Quantifying potential impacts on a basin scale might thus help to increase transparency in the discussion process and support international agreements.

### Uncertainty in the underlying data

The analysis is quantitative, which allows a sound comparison among different regions, but the numbers given should be considered as relative indicators due to the limitations and the uncertainty of the underlying data. The actual datasets on existing dams and future hydropower dams undergo continuous updates that are not necessarily reflected in the databases. Both databases represent “conservative” numbers of dams because of the underlying size limitation (existing dams larger than 15 m in dam height, future hydropower dams >1 MW in capacity), the restriction to available data that directly provide or allow manual derivation of the precise location of the dam (coordinates) and, in case of future dams, the restriction to the hydropower purpose. Many more dams can be expected that are built or planned to be constructed for purposes like irrigation, flood control or drinking water supply. Nevertheless, these data are currently the most consistent and comprehensive on the global scale that are available. Similarly, data on species ranges bear uncertainty in their timeliness and completeness. Accurate information on distribution ranges for many species (e.g. *Arapaima* spp.) remains uncertain. On the global scale, deviations from the reference scenario (details in the SI) can be considered to be small since they do not change the general pattern of the results.

## Conclusions

The present results allow those areas to be identified that deserve major attention with respect to potential conflicts between climate change mitigation (in terms of hydropower dam construction) and river ecosystem conservation. This means that evidence-based information is needed to discuss medium and long-term consequences of location options of hydropower dams at the corresponding catchment area level. Analyses on potential impacts of dam construction are not new. However, the approach presented in this study takes the (sub-)catchment and major basin scale into account, a scale that is naturally given by the connectivity of the river network, instead of a single case study neglecting upstream and downstream dam building activities. Following the analyses presented here, a ranking system could be developed for each river basin that is faced with dam construction, e.g. similarly to the WWF Orinoco River Basin 2016 Report Card^39^. This ranking system is based on the indicators about species richness and threat status and the respective interests and values of the stakeholders involved in the discussion process on dam locations. This ranking would indicate where further assessments are required before decisions about dam construction are taken, aiming to generate a common understanding of all parties involved. The analyses can help to have information on biodiversity effectively included in the decision making on dam construction too, including details on species richness and threat status. Nevertheless, prioritization of hydropower projects in a biodiversity context can only be one out of several criteria. If additional indicators for impacts of dam building were integrated this methodology would also be transferable to include further ecological, but also social and economic aspects in order to support sustainable dam construction on a river basin scale.

## Methods

### Spatial scale

Spatial analyses were conducted on the HydroBASINS **sub-catchment** level (resolution level 8)^40^. HydroBASINS uses a nested approach to delineate sub-catchments on the global scale, depending on the spatial resolution, and provides the location and area (in km^2^) of each sub-catchment. Results were aggregated on different scales dependent upon the underlying research objective: **global** scale, **latitudinal** scale, and **major basin** scale as defined by the FAO (Food and Agriculture Organization of the United Nations) and derived from HydroSHEDS (World Map of the Major Hydrological Basins: http://www.fao.org/geonetwork/srv/en/main.home). More details on the respective procedures are given below.

### Underlying data

Data for existing dams worldwide were obtained from the Global Reservoir and Dams database (GRanD)^41^. This database includes information on the coordinates of each dam, its height, and the main purpose of the reservoir (e.g. for irrigation, hydropower, flood control). For future, proposed hydropower dams, coordinates and capacity (MW) were derived from an updated version of the database developed by Zarfl *et al*.^3^. The update took place between 2015 and 2017 and includes removal of entries for future hydropower dams that have been completed in the meantime, removal of entries for which (online) references were no longer available, addition of the most recently available data for hydropower plants, and a plausibility check of the coordinates in terms of their location in relation to the respective river network. The updated database contains 3,682 future hydropower dams (in comparison to 3,700 entries in the original database), mainly with increases in entries for South America (+137) and Africa (+99) and a decrease for Asia (−238).

A comprehensive inventory of 207 extant freshwater megafauna species (≥30 kg)^18^ provides the basis for congruence analyses between dam locations and freshwater megafauna species richness. Freshwater megafauna, which include mammals (31 species), reptiles (44), amphibians (2) and fishes (130), are defined here as animals that spend their whole life or an essential part of it in fresh or brackish waters^18,19^. The contemporary geographic distribution ranges of all megafauna species have been mapped to HydroBASINS sub-catchments at resolution level 8^20,40,42^.

For each species, the conservation status according to IUCN is assigned^42^. Species assigned the threat status “Critically Endangered” (CR), “Endangered” (EN), or “Vulnerable” (VU) are integrated into the category “threatened” species. In addition, a ‘Presence’ classification is given for each species and each sub-catchment. This classification assigns a level of certainty of occurrence of the respective species in a given sub-catchment. Following the approach of earlier studies, species distribution ranges categorized as “Extant” and “Probably Extant” were considered^19,20^. In order to strengthen the results of this study by including the potential data uncertainty, the ‘Presence’ classification was used to distinguish two more scenarios depending on the “certainty” about species occurrence (see Supporting Information for details).

### Species richness within spatial overlap of existing dams and future hydropower dams

In a first step, the freshwater megafauna distribution data that assign each species to sub-catchments with conservation status and presence category have been summarized into one global database. This database was processed further with ArcGIS 10.1 and summarizes the data over the sub-catchment IDs to count total freshwater megafauna species numbers (henceforth called “species richness”) and threatened (CR, EN, VU) megafauna species numbers (1) per latitudinal band in 10° steps and (2) per sub-catchment (HydroBASINS level 8) which results in a global distribution map showing species richness.

For (1), dam numbers (existing dams (all purposes) and future hydropower dams) were counted per latitudinal band. For each latitudinal band, total land area was determined by summing up sub-catchment areas within the respective latitude. This allowed normalizing dam and species numbers to the area available for dam construction and freshwater species distribution range.

For (2), the spatial overlap of existing dams and future hydropower dams was analysed by assigning the dam data to the HydroBASINS sub-catchments and thus to the respective species richness of each sub-catchment. Sub-catchments were categorized according to the number of freshwater megafauna species that occur within the respective sub-catchment. Sub-catchments containing the same species number were summarized in adding up (1) the respective total surface area, (2) surface area containing existing dams, (3) surface area containing future hydropower dams, and (4) the surface area containing existing dams and/or future hydropower dams.

### Spatial overlap between hydropower dams and sub-catchments rich in threatened megafauna species

In addition to the total number of freshwater megafauna species (species richness) the fraction of threatened species was calculated for each sub-catchment. Species richness and the proportion of threatened species were combined into choropleth maps. For these maps, one colour indicator per characteristic was subdivided into four categories resulting in a matrix of 16 ‘richness-threat’ subcategories in total. Species richness was subdivided according to the distribution of numbers of sub-catchments (HydroBASINS level 8) per species number (1 to 23 species on the global scale) in 10-percentile, mean and 90-percentile in analogy to the general methodology in hydrology describing river discharge with Q_10_, Q_mean_, Q_90_. This division accounts for the lowest (poor) and highest (rich) number of species that occur in less than 10% of the sub-catchments. Since species numbers can be integers only the number that is the next closest to the respective percentile is chosen (Fig. S2.i). With this, the four categories contain on the global scale 1 species, 2 to 4 species, 5 to 8 species, and more than 8 species. In order to analyse the sensitivity of choosing this type of categorization three further approaches in selecting category boundaries have been analysed (Fig. S2ii–S2iv). Subdivision of the fraction of threatened freshwater megafauna species was equally distributed into up to 25% of the species threatened, up to 50% threatened, up to 75% threatened and more than 75% threatened.

In a next step, the number of existing and future hydropower dams per richness-threat category was derived from the overview on sub-catchments using the Field Calculator function of ArcGIS. All sub-catchments containing the same richness-threat category were summarized in adding up (1) the respective area in total, (2) the number of all existing hydropower dams within each richness-threat category, and (3) the number of all future hydropower dams within each richness-threat category. Based on these data, dam “densities” were calculated per 100,000 km^2^ for each richness-threat category.

### Dams with highest potential impact on freshwater megafauna within a major basin

The procedure described above for categorizing sub-catchments according to their species richness and share in threatened species was repeated for major basins. The same methodology as above was applied for each specific major basin to subdivide species richness data into four sub-categories from poor to rich (category boundaries are: 10-percentile, mean, 90-percentile). Due to different species richness in the basins absolute category boundaries may differ between the global and basin scale analysis. Data on species richness, share in threatened species and richness-threat category on a sub-catchment resolution were then assigned to the existing dams and future hydropower dams located in the respective sub-catchment. Based on this characterization those dams located in sub-catchments of the major basin with the highest species richness and richness in threatened species can be identified. Results for six selected major basins (Amazon, Balkans as the combination of ‘Adriatic Sea – Greece – Black Sea Coast’ and ‘Danube’, Congo, Ganges-Brahmaputra, Mekong, Yangtze) were presented in choropleth maps.

## Supplementary information

SUPPORTING INFORMATION: Future large hydropower dams impact global freshwater megafauna

## Data Availability

Dam data are available for download from the Global Dam Watch homepage http://globaldamwatch.org/data/. All other spatial data analysed here are available following the respective references.

## References

[1] The World Bank. Database World Development Indicators, https://data.worldbank.org (2018).

[2] The World Bank. *Atlas of Sustainable Development Goals 2018: World Development Indicators* (2018).

[3] Zarfl C, Lumsdon AE, Berlekamp J, Tydecks L, Tockner K (2015). A global boom in hydropower dam construction. Aquat. Sci..

[4] EuroNatur. Verdammt gefährdet. *EuroNatur Magazin* **2**, 10–13, https://www.euronatur.org/fileadmin/docs/magazin/EuroNatur_Magazin_2-2018.pdf (2018).

[5] Berga L (2016). The role of hydropower in climate change mitigation and adaptation: A review. Engineering.

[6] International Commission on Large Dams. *World Register of Dams*, https://www.icold-cigb.org/GB/world_register/world_register_of_dams.asp (2011).

[7] Richter BD, Postel S, Revenga C, Lehner B, Churchill A (2010). Lost in development’ s shadow: The downstream human consequences of dams. Water Altern..

[8] Constantine, J. A., Dunne, T., Ahmed, J., Legleiter, C. & Lazarus, E. D. Sediment supply as a driver of river meandering and floodplain evolution in the Amazon Basin. *Nat*. *Geosci*. **7**, 899–903 (2014).

[9] Zarfl C, Lucía A (2018). The connectivity between soil erosion and sediment entrapment in reservoirs. Curr. Opin. Environ. Sci. Heal..

[10] Gibson, L., Wilman, E. N. & Laurance, W. F. How green is ‘green’ energy? *Trends Ecol*. *Evol*. **32**, 922–935 (2017). 10.1016/j.tree.2017.09.007 29074270

[11] O’Neil JM, Davis TW, Burford MA, Gobler CJ (2012). The rise of harmful cyanobacteria blooms: The potential roles of eutrophication and climate change. Harmful Algae.

[12] Lerer LB, Scudder T (1999). Health impacts of large dams. Environ. Impact Assess. Rev..

[13] Johnson PTJ, Olden JD, Zanden MJ (2008). Vander. Dam invaders: impoundments facilitate biological invasions into freshwaters. Front. Ecol. Environ..

[14] Nilsson C, Reidy CA, Dynesius M, Revenga C (2005). Fragmentation and flow regulation of the world’s large river systems. Science.

[15] Reid, A. J. *et al*. Emerging threats and persistent conservation challenges for freshwater biodiversity. *Biol*. *Rev*. **94**, 849-873 (2018). 10.1111/brv.12480 30467930

[16] Winemiller, K. O. *et al*. Balancing hydropower and biodiversity in the Amazon, Congo, and Mekong. *Science* **351**, 128-129 (2016). 10.1126/science.aac7082 26744397

[17] Secretariat of the Convention on Biological Diversity. *Nagoya Protocol*, https://www.cbd.int/abs/ (2011).

[18] He F (2017). Disappearing giants: a review of threats to freshwater megafauna. Wiley Interdiscip. Rev. Water.

[19] Carrizo SF (2017). Freshwater megafauna: Flagships for freshwater biodiversity under threat. Bioscience.

[20] He, F. *et al*. Freshwater megafauna diversity: Patterns, status and threats. *Divers*. *Distrib*. **24**, 1395– 1404 (2018).

[21] Liermann CR, Nilsson C, Robertson J, Ng RY (2012). Implications of dam obstruction for global freshwater fish diversity. Bioscience.

[22] van Puijenbroek P, Buijse A, Kraak M, Verdonschot P (2019). Species and river specific effects of river fragmentation on European anadromous fish species. River Res. Appl..

[23] He, F. *et al*. The global decline of freshwater megafauna. *Glob*. *Chang*. *Biol*. **25**, 3883– 3892 (2019). 10.1111/gcb.14753 31393076

[24] Bartle, A. & Usher, S. *The International Journal on Hydropower and Dams – World Atlas and Industry Guide*. (Aqua-Media International Ltd. UK, 2015).

[25] Zuo WY, Smith FA, Charnov EL (2013). A life‐history approach to the late Pleistocene megafaunal extinction. Am. Nat..

[26] Intralawan A, Wood D, Frankel R, Costanza R, Kubiszewski I (2018). Tradeoff analysis between electricity generation and ecosystem services in the Lower Mekong Basin. Ecosyst. Serv..

[27] Pereira LS (2018). Non-native species in reservoirs: how are we doing in Brazil?. Hydrobiologia.

[28] Zheng S (2018). Riverbed erosion of the final 565 kilometers of the Yangtze River (Changjiang) following construction of the Three Gorges Dam. Sci. Rep..

[29] Remo JWF, Ickes BS, Ryherd JK, Guida RJ, Therrel MD (2018). Assessing the impacts of dams and levees on the hydrologic record of the Middle and Lower Mississippi River, USA. Geomophology.

[30] Wu JM (2015). Drastic decline in spawning activity of Chinese sturgeon Acipenser sinensis Gray 1835 in the remaining spawning ground of the Yangtze River since the construction of hydrodams. J. Appl. Ichthyol..

[31] Zhuang P (2016). New evidence may support the persistence and adaptability of the near-extinct Chinese sturgeon. Biol. Conserv..

[32] Dunn FE (2018). Projections of historical and 21st century fluvial sediment delivery to the Ganges-Brahmaputra-Meghna, Mahanadi, and Volta deltas. Sci. Total Environ..

[33] Maavara T, Parsons CT, Ridenour C, Stojanovic S, Dürr HH (2015). Global phosphorus retention by river damming. Proc. Natl. Acad. Sci. USA.

[34] Maavara, T., Lauerwald, R., Regnier, P. & Van Cappellen, P. Global perturbation of organic carbon cycling by river damming. *Nat*. *Commun*. **8**, 15347 (2017). 10.1038/ncomms15347 PMC5442313 28513580

[35] Grill G (2015). An index-based framework for assessing patterns and trends in river fragmentation and flow regulation by global dams at multiple scales. Environ. Res. Lett..

[36] Poff, N. L. & Schmidt, J. C. How dams can go with the flow. *Science* **353**, 1099–1100 (2016). 10.1126/science.aah4926 27609876

[37] Harrison Ian J., Green Pamela A., Farrell Tracy A., Juffe-Bignoli Diego, Sáenz Leonardo, Vörösmarty Charles J. (2016). Protected areas and freshwater provisioning: a global assessment of freshwater provision, threats and management strategies to support human water security. Aquatic Conservation: Marine and Freshwater Ecosystems.

[38] International Hydropower Association. Can these unlock hydro potential in the Balkans? https://www.hydropower.org/blog/can-these-unlock-hydro-potential-in-the-balkans (2017).

[39] WWF. *Orinoco River Basin Report Card*, https://www.worldwildlife.org/pages/orinoco-river-basin-report-card (2016).

[40] Lehner B, Grill G (2013). Global river hydrography and network routing: Baseline data and new approaches to study the world’s large river systems. Hydrol. Process..

[41] Lehner B (2011). High-resolution mapping of the world’s reservoirs and dams for sustainable river-flow management. Front. Ecol. Environ..

[42] IUCN. The IUCN Red List of Threatened Species. Version 2016-3, http://www.iucnredlist.org (2016).

