# Supplementary material for: Future large hydropower dams impact global freshwater megafauna
Source: Sci Rep. 2019 Dec 6;9:18531. doi: 10.1038/s41598-019-54980-8 (PMC6898151; doi:10.1038/s41598-019-54980-8)
Supplement: Supplementary file 1 — SUPPORTING INFORMATION: Future large hydropower dams impact global freshwater megafauna [file 41598_2019_54980_MOESM1_ESM.pdf]

SUPPORTING INFORMATION

Future large hydropower dams impact global freshwater megafauna

Christiane Zarfl[1,*], Jürgen Berlekamp[2], Fengzhi He[3,4,5], Sonja C. Jähnig[3], William Darwall[6], Klement Tockner[3,4,7]

[1] Center for Applied Geosciences, Eberhard Karls University of Tübingen, Hölderlinstr. 12, 72074 Tübingen, Germany.

[2] Institute of Environmental Systems Research, University of Osnabrück, Barbarastraße 12, 49076 Osnabrück, Germany.

[3] Department of Ecosystem Research, Leibniz-Institute of Freshwater Ecology and Inland Fisheries, Müggelseedamm 310, 12587 Berlin, Germany.

[4] Department of Biology, Chemistry and Pharmacy, Freie Universität Berlin, Altensteinstraße 6, 14195 Berlin, Germany.

[5] School of Geography, Queen Mary University of London, London E1 4NS, UK

[6] Freshwater Biodiversity Unit, IUCN Global Species Programme, The David Attenborough Building, Pembroke Street, Cambridge, CB2 3QZ, United Kingdom.

[7] Austrian Science Fund, Sensengasse 1, 1090 Vienna, Austria.

[*] To whom correspondence should be addressed. Email: christiane.zarfl@uni-tuebingen.de, phone: +49 (0)7071-29-76076

Species distribution range scenarios. 'Presence' classification of the megafauna species data provided by IUCN was used to distinguish three scenarios depending on the "certainty" about species occurrence. While for clarity reasons, results of the reference scenario only are given in the main manuscript, results presented in the Supporting Information contain also details on the variability in the results due to different species presence information:

- Scenario 1. 'Presence' = "Extant": known or very likely existence of the species in the sub-catchment based on records of the last 30 years, corresponds to the **minimum** area inhabited by the respective species.
- Scenario 2. 'Presence' = "Extant" or "Probably Extant": most probable existence of the species in the sub-catchment. (Note: According to IUCN the category "probably extant" will be phased out in the future but is currently still contained in the data.) **Reference scenario**, also applied by IUCN.
- Scenario 3. 'Presence' = "Extant", "Probably Extant", "Possibly Extant" or "Presence Uncertain": includes sub-catchments in which the species occurred most likely, but recent records are missing or records require verification and are highly uncertain. This scenario corresponds to the **maximum** possible area inhabited by the respective species.

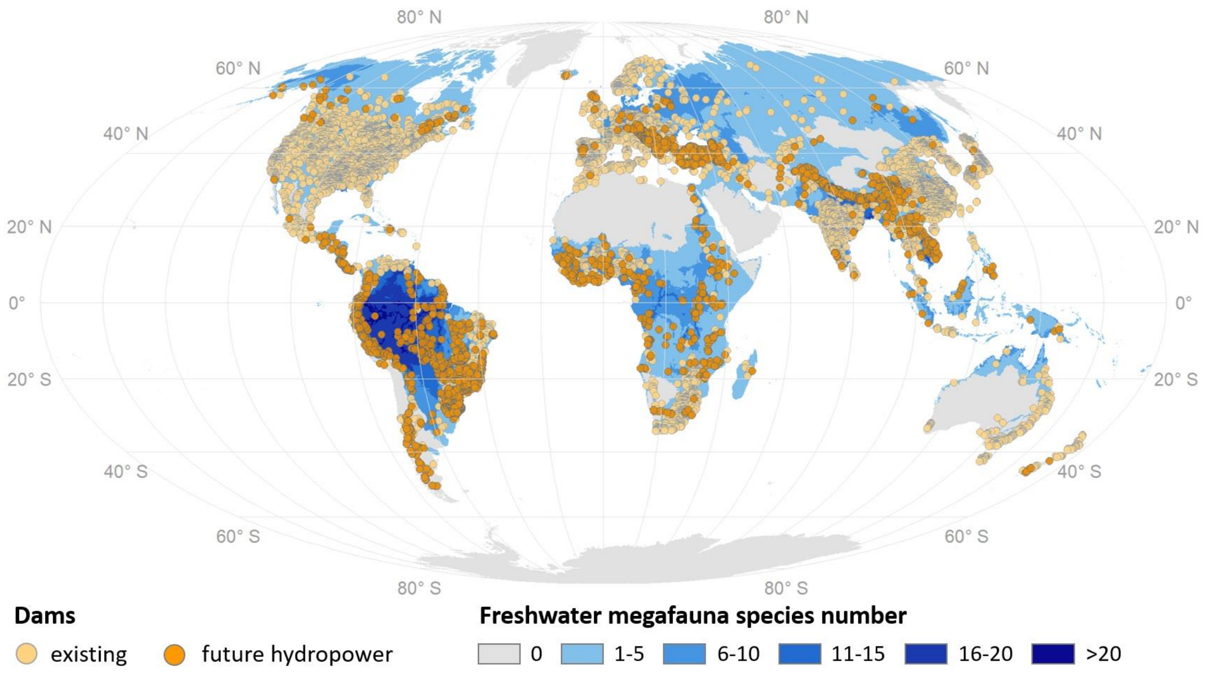

Fig. S1 Overlap of existing dams and future hydropower dams with numbers of freshwater megafauna species per sub-catchment on a global scale.

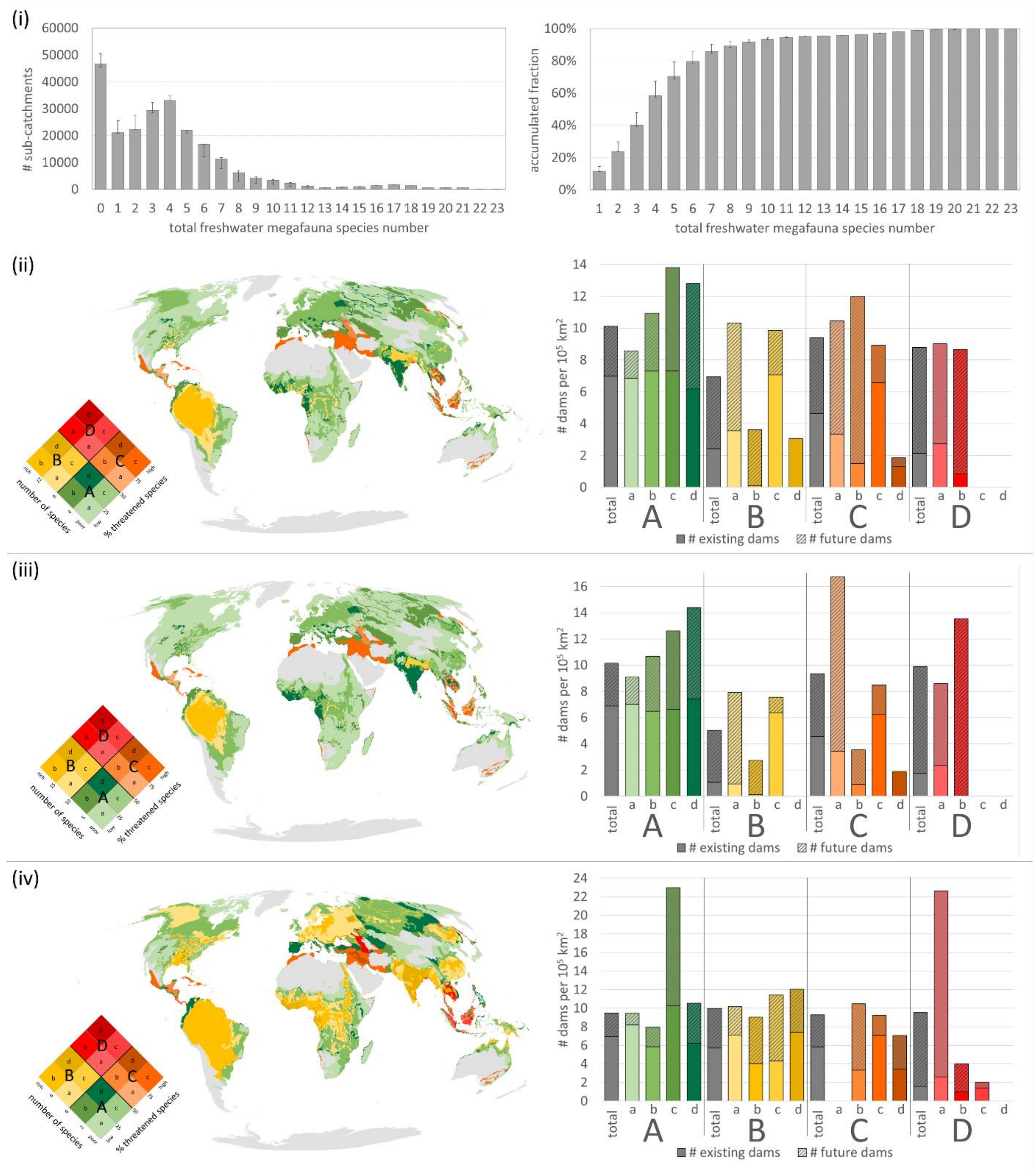

Fig. S2 Underlying analysis for derivation of sub-categories for species richness. (**i**) Distribution of sub-catchment numbers per total freshwater megafauna species number on a global scale as absolute numbers (left) and accumulated percentage (right). Error bars give the range for the three scenarios, which vary in species occurrence data. (**ii**) – (**iv**) Results of different species richness categorizations reflected in choropleth maps of sub-catchments (HydroBASINS level 8) according to species richness and threat status (left) and "density" of dams per 10^5 km^2 in different sub-catchments according to richness-threat categories (right), both based on the reference scenario for species occurrence. Species richness categories have been selected as follows: (**ii**) 5% of the sub-catchments represent highest species richness (> 12 species) and the remaining 95% of sub-catchments with lower species richness are classified with equal distances (1-4 species; 5-8 species; 8-11 species). (**iii**) equal distances between absolute species numbers (1-5 species: 70% of sub-catchments; 6-10 species: 23%; 11-15 species: 3%; > 15 species: 4%). (**iv**) equal size fractions in terms of sub-catchment numbers of each category (1-2 species; 3-4 species; 5-6 species; > 6 species). This gives very similar results in comparison to the selected methodology as described in the main text.

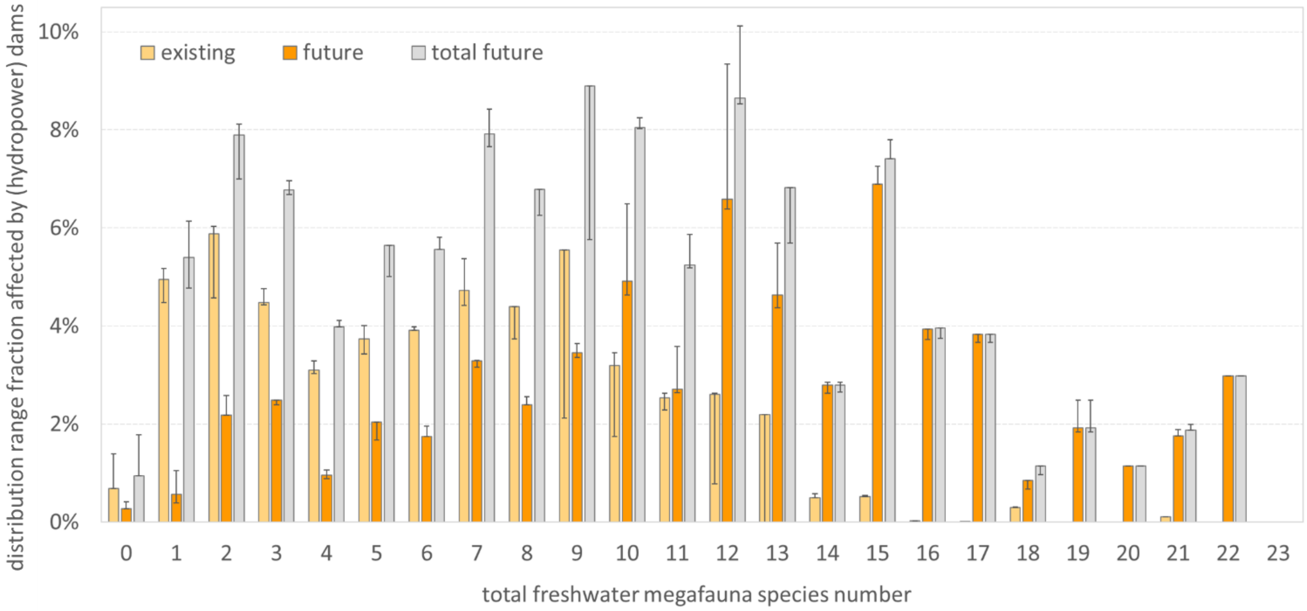

Fig. S3 Fractions of freshwater megafauna distribution ranges per freshwater megafauna species number that are directly affected by existing dams and by future hydropower dams. Please note that the total fraction of the distribution range affected in the future might be smaller than the sum of the range fractions of existing and future dams since future hydropower dams partly fall into the same sub-catchment as existing ones and this area is not counted twice. Error bars result from scenarios in species occurrence.

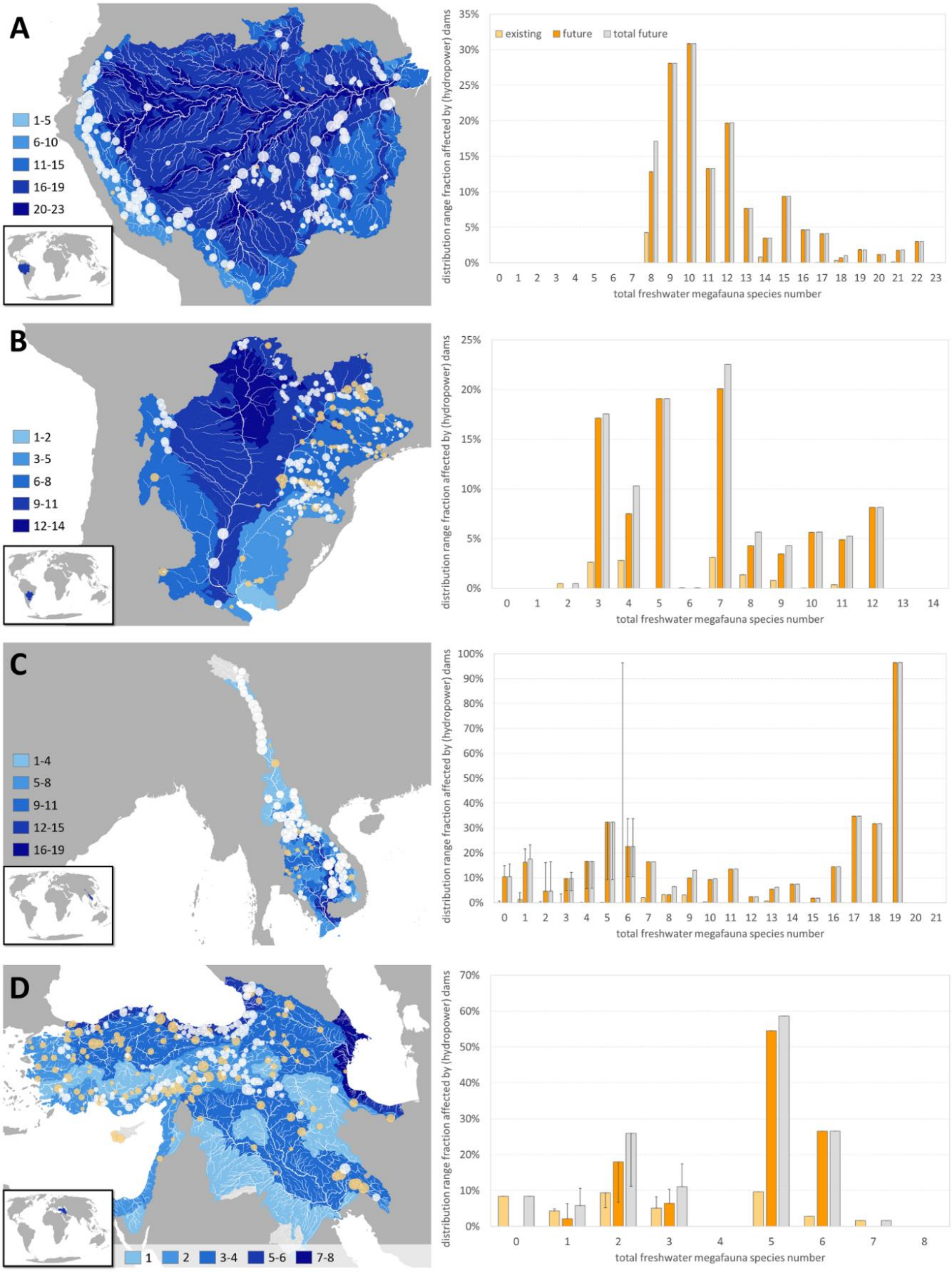

Fig. S4.1 Dams in selected major basins and their overlap with species richness of freshwater megafauna. Left: Location of existing dams (yellow) and future hydropower dams (white) and distribution ranges of species richness (blue shading). Size of the dots indicates size of the dam (existing: dam height in m; future: capacity in MW – not scaled). Right: Distribution range fractions that are or will be potentially affected by dam construction per species number category. Error bars give the range for the three scenarios, which vary in species occurrence data. If error bars are missing presence data for the species are the same in all three scenarios. **A**: **Amazon** – 8 existing dams, 368 future hydropower dams. **B**: **La Plata** – 80 existing, 409 future. **C**: **Mekong** – 20 existing, 120 future. **D: Black Sea, South Coast & Caspian Sea, South West Coast & Mediterranean Sea, East Coast & Tigris-Euphrates** – 132 existing, 286 future.

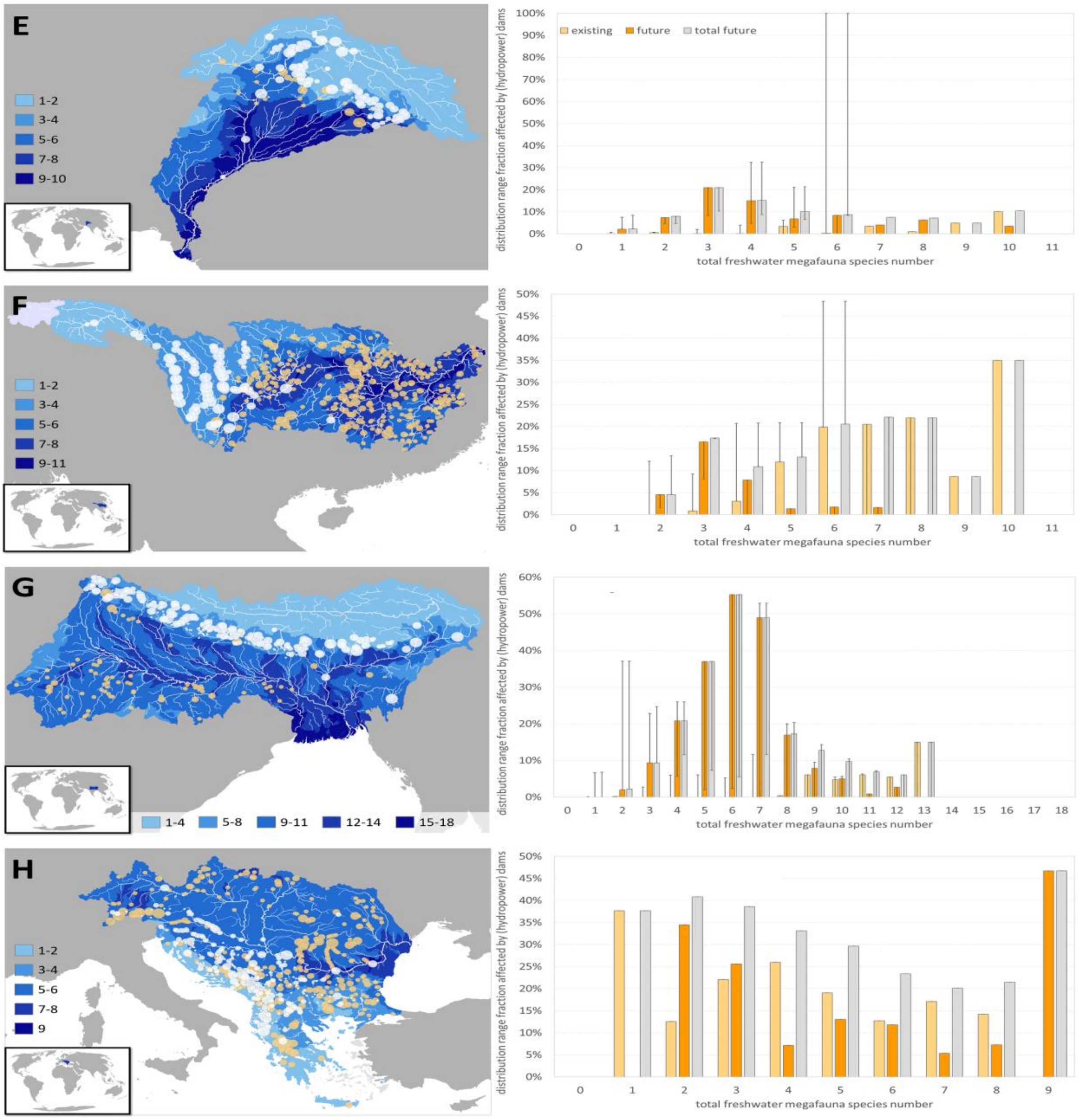

Fig. S4.2 Dams in selected major basins and their overlap with species richness of freshwater megafauna (see figure caption to first part of this Figure). **E**: **Indus** – 31 existing dams, 104 future hydropower dams. **F**: **Yangtze** – 346 existing, 84 future. **G**: **Ganges-Brahmaputra** – 83 existing, 396. **H**: **Balkan** region – 270 existing, 611 future.

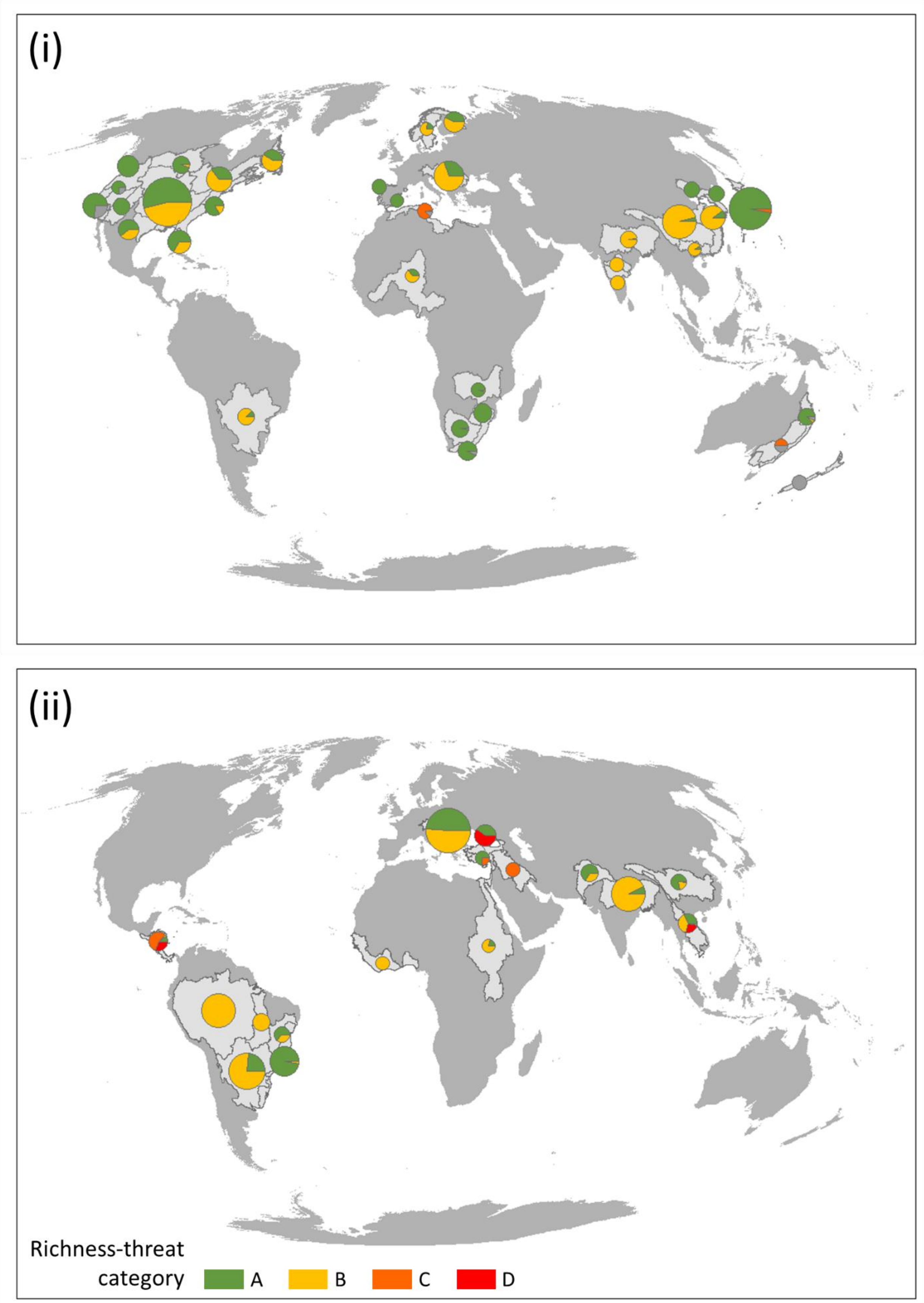

Fig. S5 Major basins with (**i**) more than 100 existing dams or (**ii**) more than 100 future hydropower dams and the respective share in overlap with megafauna distribution ranges according to their richness-threat category. Size of the pie chart indicates the number of dams per major basin.

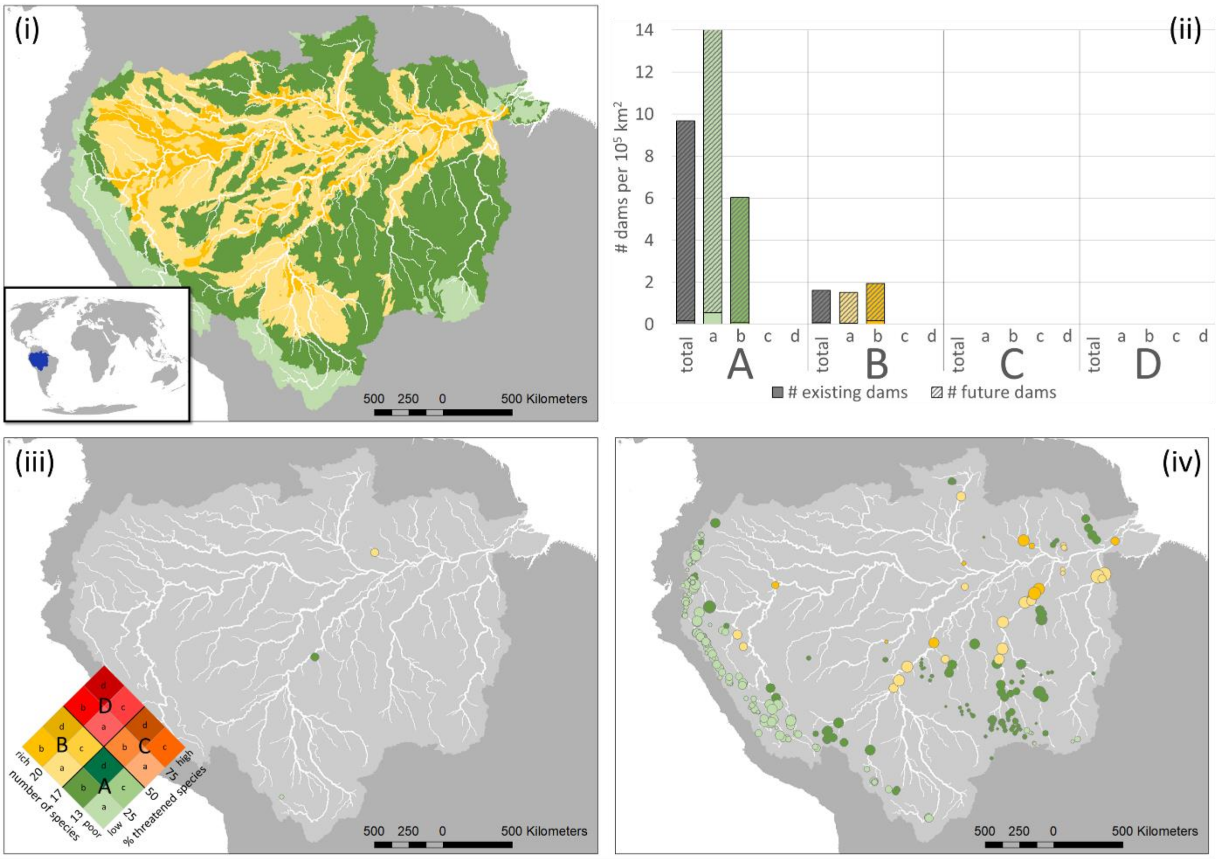

Fig. S6.1 Overlap of dams with freshwater megafauna species richness and share in threatened species for the **Amazon** basin. *Top*: (**i**) Choropleth map of sub-catchments (HydroBASINS level 8) according to species richness and threat status (species presence reference scenario). (**ii**) "Density" of dams per 10^5 km^2 in different sub-catchments according to richness-threat categories. Existing dams: bold colour; Future hydropower dams: striped colour. *Bottom*: Location of (**iii**) existing dams and (**iv**) future hydropower dams and their individual categorization according to species richness and proportion of threatened species. Size of the dots indicates size of the dam (existing: dam height in m; future: capacity in MW – not scaled). *Colour code:* Green (A): low richness (≤ 17 species), low share in threatened species (≤ 50%); Yellow (B): high richness (> 17 species), low share in threatened species; Orange (C): low richness, high share in threatened species (> 50%); Red (D): high richness, high share in threatened species. For more details, please see legend in subfigure (iii).

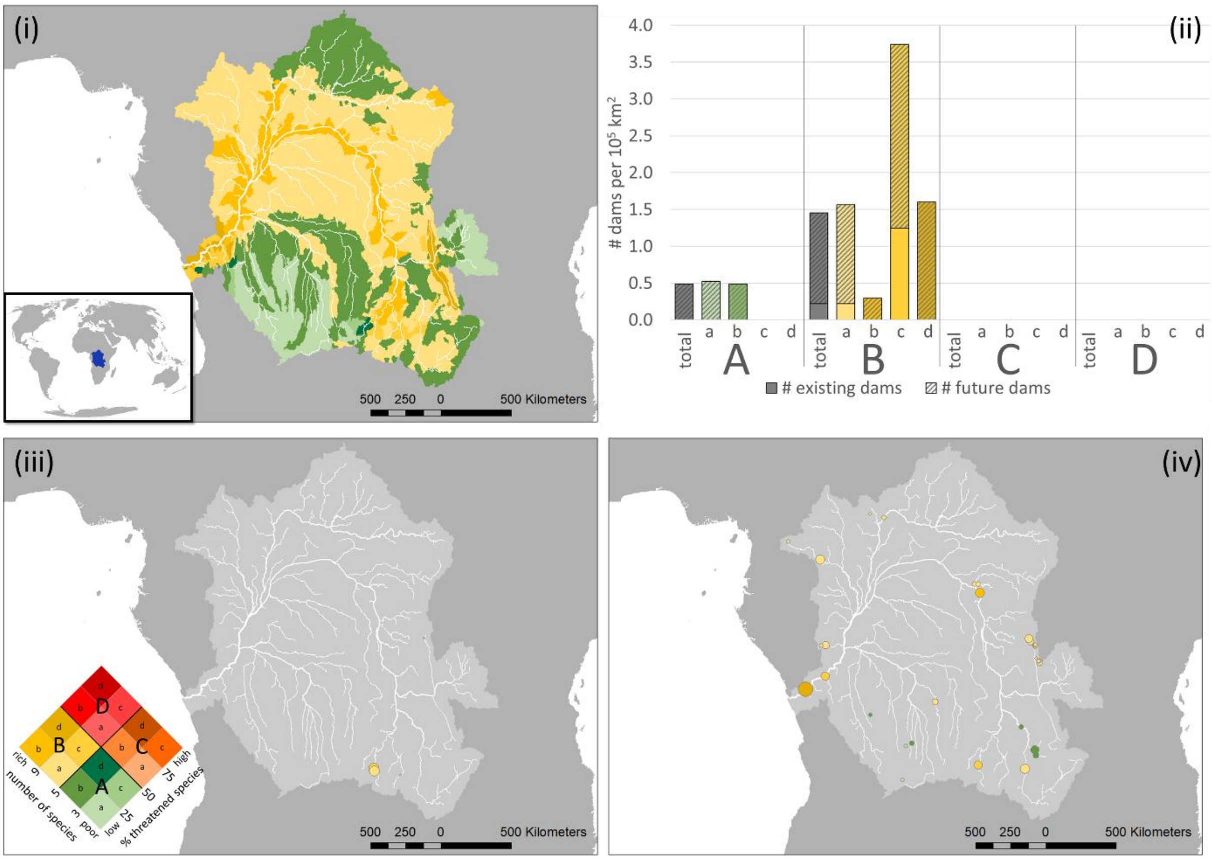

Fig. S6.2 Overlap of dams with freshwater megafauna species richness and share in threatened species for the **Congo** basin. *Colour code:* Green (A): low richness (≤ 5 species), low share in threatened species (≤ 50%); Yellow (B): high richness (> 5 species), low share in threatened species; Orange (C): low richness, high share in threatened species (> 50%); Red (D): high richness, high share in threatened species. For more details, please see legend in subfigure (**iii**) and figure caption of Figure S6.1.

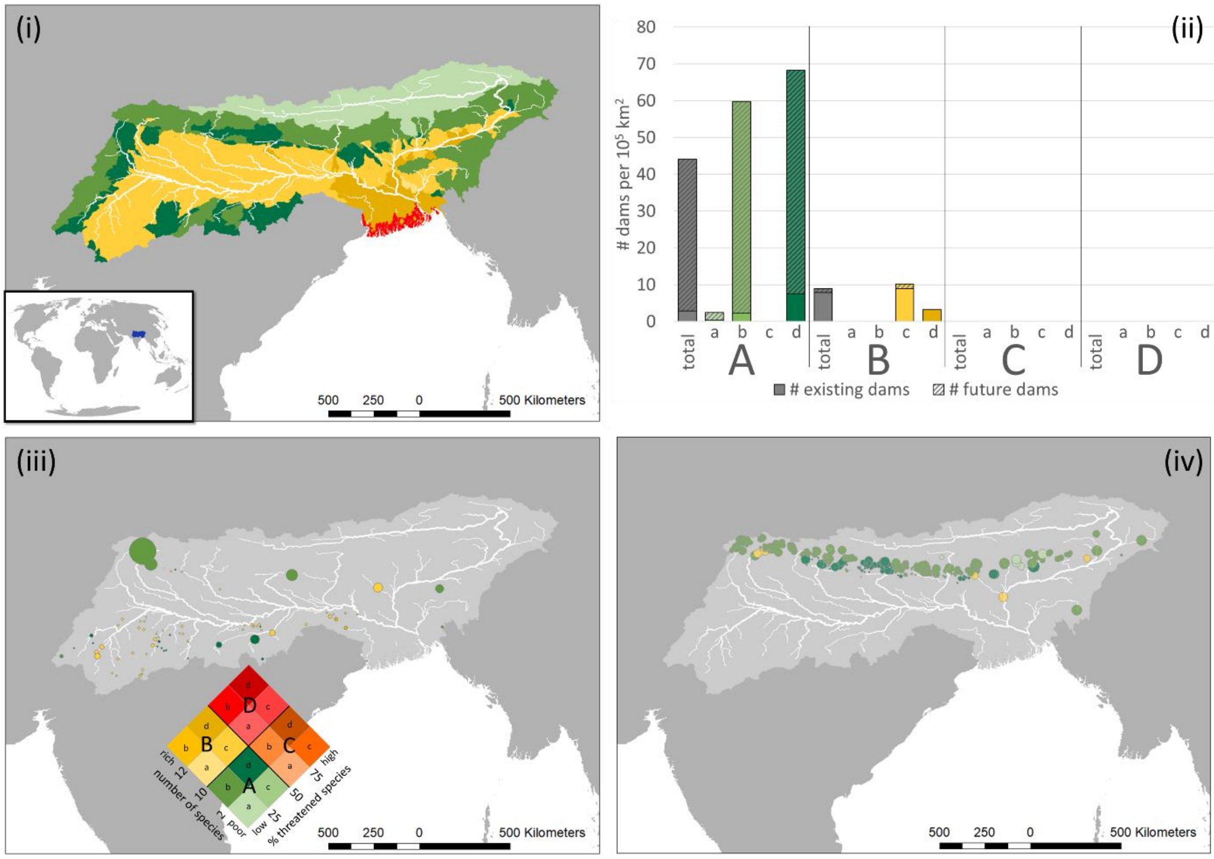

Fig. S6.3 Overlap of dams with freshwater megafauna species richness and share in threatened species for the **Ganges-Brahmaputra** basin. *Colour code:* Green (A): low richness (≤ 10 species), low share in threatened species (≤ 50%); Yellow (B): high richness (> 10 species), low share in threatened species; Orange (C): low richness, high share in threatened species (> 50%); Red (D): high richness, high share in threatened species. For more details, please see legend in subfigure (**iii**) and figure caption of Figure S6.1.

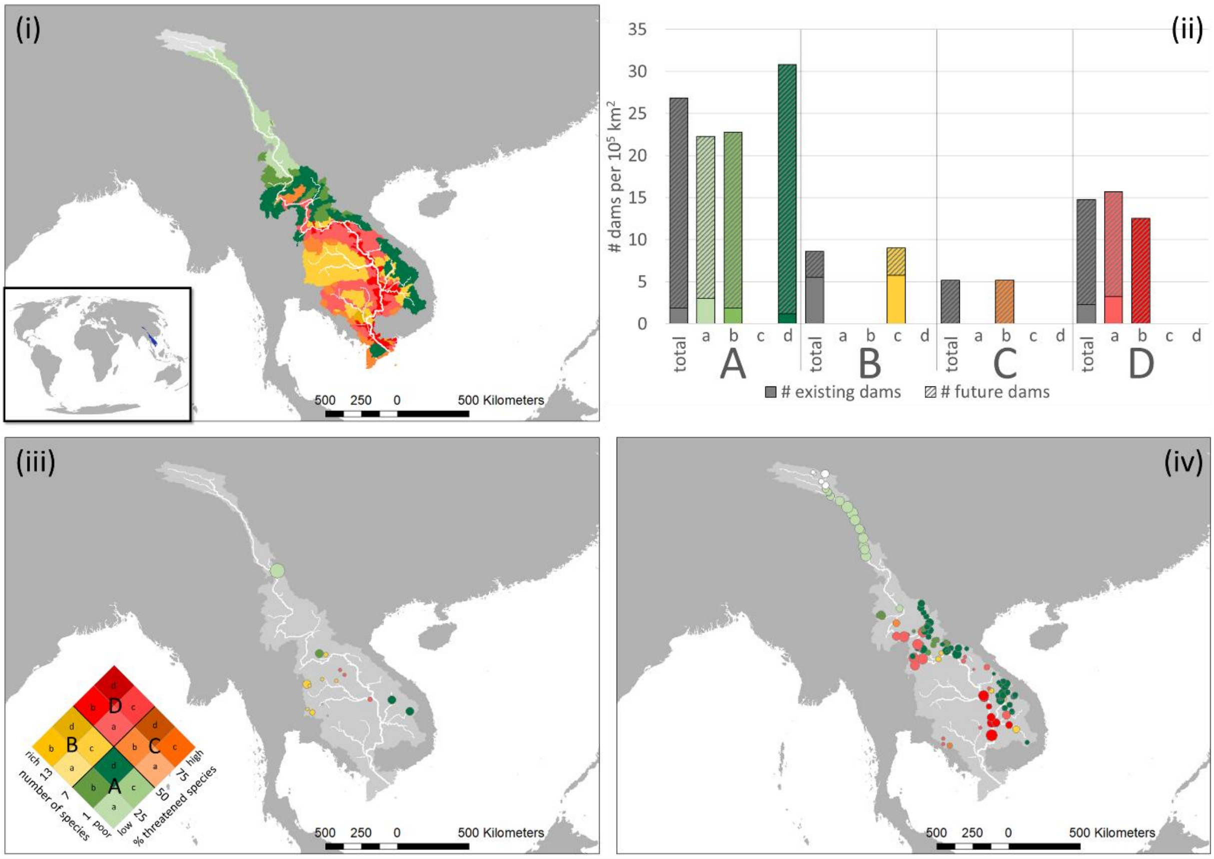

Fig. S6.4 Overlap of dams with freshwater megafauna species richness and share in threatened species for the **Mekong** basin. *Colour code:* Green (A): low richness (≤ 7 species), low share in threatened species (≤ 50%); Yellow (B): high richness (> 7 species), low share in threatened species; Orange (C): low richness, high share in threatened species (> 50%); Red (D): high richness, high share in threatened species. For more details, please see legend in subfigure (**iii**) and figure caption of Figure S6.1.

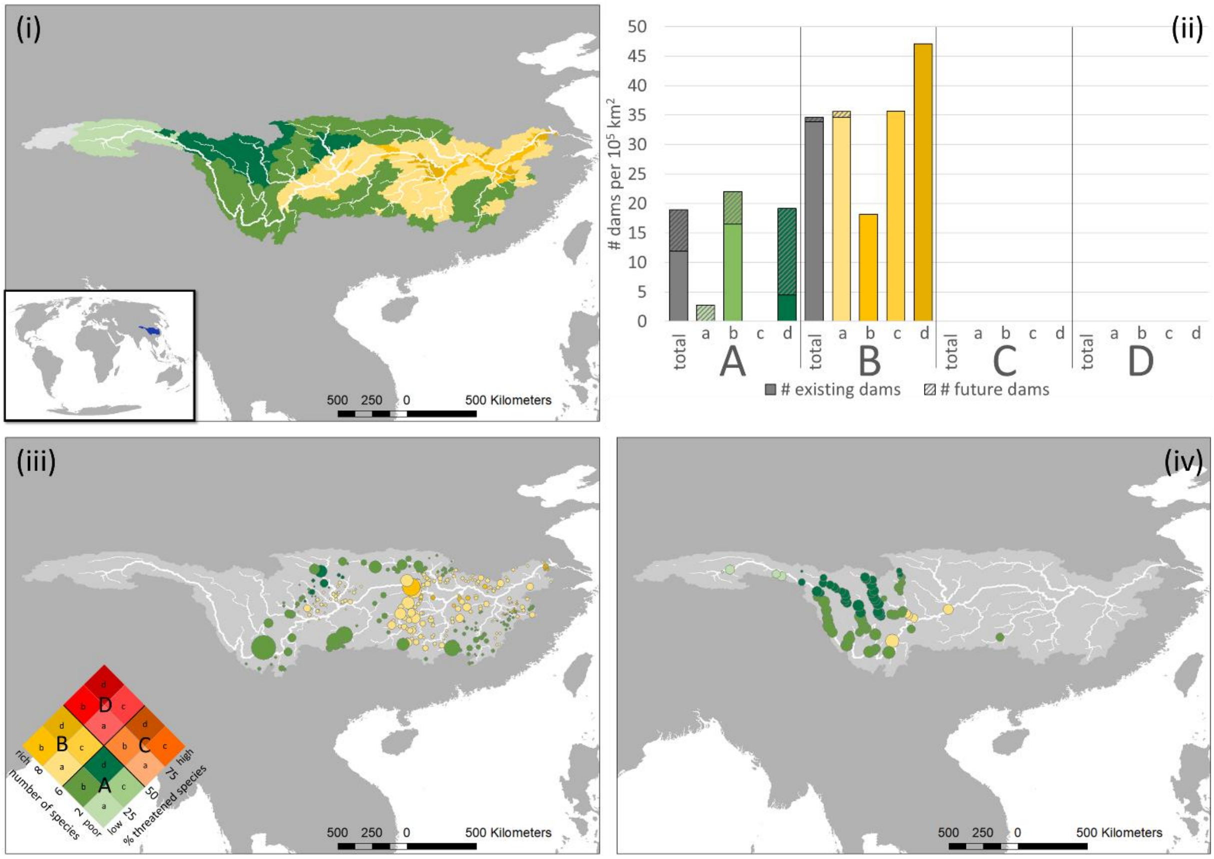

Fig. S6.5 Overlap of dams with freshwater megafauna species richness and share in threatened species for the **Yangtze** basin. *Colour code:* Green (A): low richness (≤ 6 species), low share in threatened species (≤ 50%); Yellow (B): high richness (> 6 species), low share in threatened species; Orange (C): low richness, high share in threatened species (> 50%); Red (D): high richness, high share in threatened species. For more details, please see legend in subfigure (**iii**) and figure caption of Figure S6.1.

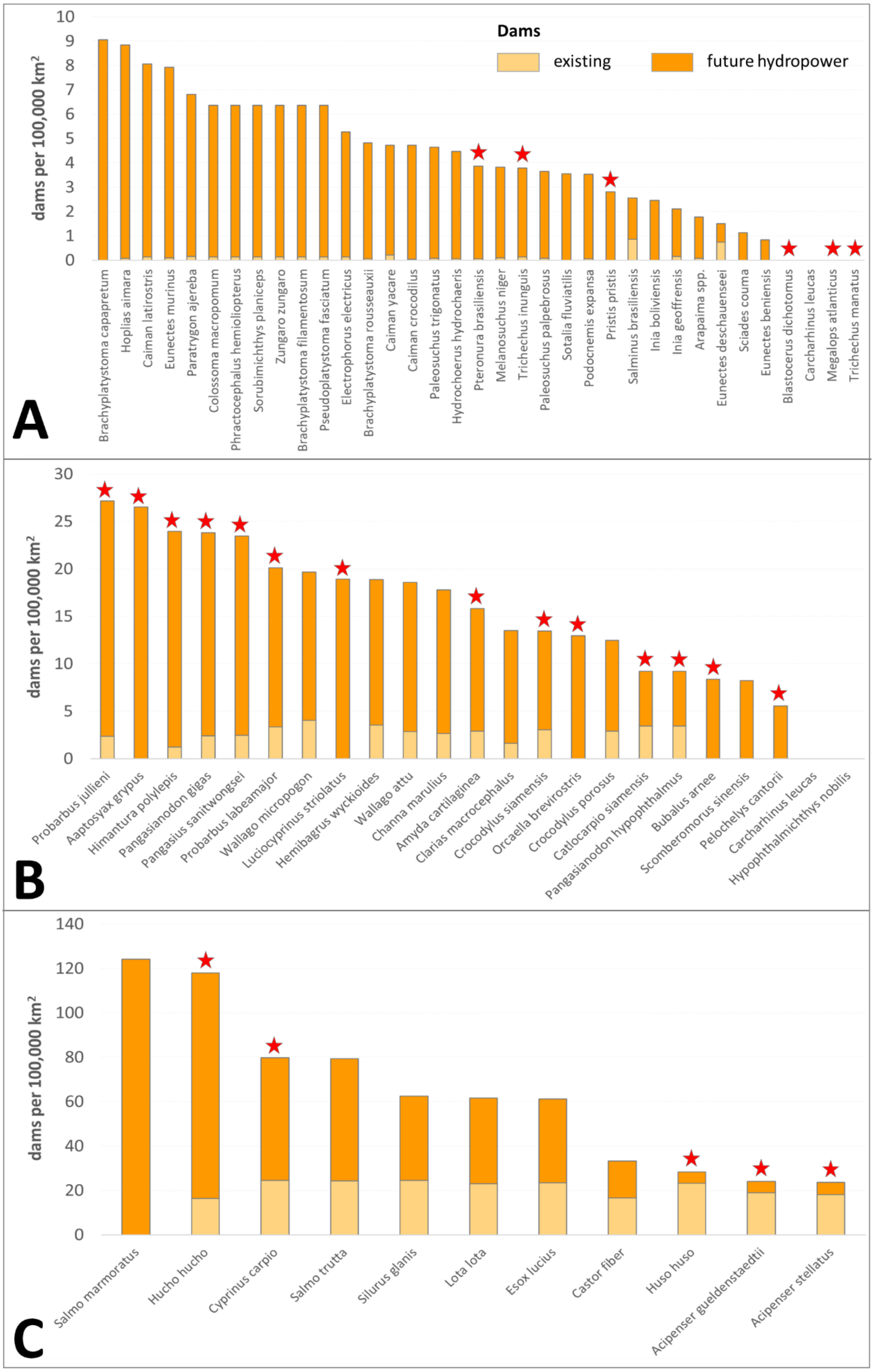

Fig. S7 Density (number per 100,000 km²) of existing dams and future hydropower dams in distribution ranges of freshwater megafauna species in three selected major basins: **A** – Amazon; **B** – Mekong; **C** – Balkan region. Red stars indicate threatened species according to IUCN Red List Categories.
